# Shallow Grooves Detection Processed by Controllable Electrolyte Distribution Electrochemical Machining (CED-ECM) Method Based on a Pseudo-Multimodal Wavelet Network (PMW-YOLO) for Lightweight Instance Segmentation

**DOI:** 10.3390/mi17080875

**Published:** 2026-07-23

**Authors:** Jing Zhao, Wanting Wei, Haotian Zheng, Qiyuan Cao, Limei Ma, Jiankang Wang

**Affiliations:** 1 School of Engineers, Beijing Institute of Petrochemical Technology, Qingyuan North Road No. 19, Daxing District, Beijing 102617, China; 2 Academy of Artificial Intelligence, Beijing Institute of Petrochemical Technology, Qingyuan North Road No. 19, Daxing District, Beijing 102617, China

**Keywords:** shallow grooves detection, controllable electrolyte distribution electrochemical machining (CED-ECM), pseudo-multimodal wavelet network (PMW-YOLO), lightweight instance segmentation, automated groove inspection

## Abstract

Controllable Electrolyte Distribution Electrochemical Machining (abbreviated as CED-ECM) is a novel ECM method for shallow groove (with a depth of about several μm to tens μm) fabrication on precision metal surfaces. Rapid and accurate detection of CED-ECM-processed grooves is essential for quality control and automated production cycles. However, these grooves exhibit minute scale, irregular morphology, and low contrast against the metallic background, while grayscale microscopic imaging provides only single-channel information. To address these challenges, this research proposes a lightweight instance segmentation network based on pseudo-multimodal wavelet network (abbreviated as PMW-YOLO). First, a pseudo-multimodal channel fusion strategy expands single grayscale images into three complementary channels: original grayscale, CLAHE-enhanced grayscale, and multiscale Sobel gradients. This design explicitly injects illumination robustness and edge priors without additional acquisition cost. Second, a Discrete Wavelet Transform-based downsampling module, termed DWTDown, is integrated into the backbone to preserve high-frequency edge details while reducing model parameters and GFLOPs. Ablation studies further investigate the contributions of an Efficient Multiscale Attention module, a boundary-aware mask loss, and data-centric augmentation strategies. Experiments on an in-house CED-ECM dataset validate the effectiveness of PMW-YOLO for automated groove inspection.

## 1. Introduction

Controllable Electrolyte Distribution Electrochemical Machining (CED-ECM) is an ECM method for fabricating shallow grooves on precision metal surfaces, with groove depths typically ranging from several micrometers to tens of micrometers [1,2]. As a non-contact process that involves no cutting force and is not limited by material hardness, ECM plays an important role in aerospace components, precision molds, and functional surface textures [3,4]. CED-ECM confines the electrolyte within a restricted medium such as a porous solid ball, thereby controlling electrolyte distribution and fabricating shallow grooves or textures on precision metal surfaces without stray corrosion [5,6]. Related Micromachines studies have also reported electrochemical micromachining routes for micro-groove fabrication, highlighting the relevance of micro-groove quality assessment in this field [7]. In this process, groove morphology, scale, and spatial distribution directly affect the service performance of functional surfaces. Rapid and accurate inspection of processed grooves is therefore essential for process quality control and automated production feedback. At present, CED-ECM-processed groove inspection still mainly relies on manual microscopic observation and offline measurement, which are inefficient, subjective, and difficult to integrate online. This has become one of the bottlenecks limiting the wider application of CED-ECM.

Automating CED-ECM processed-processed groove inspection is constrained by four coupled difficulties. First, these processed grooves are small and morphologically irregular, with weak textures and locally discontinuous boundaries that make instance localization and mask segmentation difficult [8]. Second, reflections from the metal surface, CED-ECM-processed grooves, and background grayscale fluctuations reduce the contrast between CED-ECM-processed grooves and the substrate, increasing the risk of missed detections for low-contrast targets. Third, the available images are mainly single-channel grayscale micrographs, so the network cannot directly use color, spectral, or multimodal cues. Fourth, CED-ECM sample preparation and pixel-level instance annotation are costly, leaving only a limited number of high-quality labeled samples for training. Therefore, this task is not merely a feature-enhancement problem in general surface-defect inspection. It is a lightweight instance segmentation problem jointly constrained by small target morphology, low contrast, single-channel imaging, and small-sample training.

In recent years, deep learning has substantially advanced industrial surface inspection. Semantic segmentation networks such as FCN, U-Net, DeepLab, and SegFormer [9,10,11,12] and two-stage instance segmentation methods such as Mask R-CNN [13] can achieve high segmentation accuracy. However, their parameter counts and computational costs are often too high for real-time deployment on production-line edge devices. One-stage methods represented by the YOLO family [14] balance speed and accuracy and have been widely applied to surface defect inspection in steel, metal, PCB, and micro-defect images [15,16,17,18,19]. Many variants further improve accuracy using SE, coordinate attention, and efficient multiscale attention (EMA) mechanisms [20,21,22]. However, most of these improvements have been validated on public datasets such as NEU-DET and GC10-DET, which contain thousands of images. Their central problem is usually insufficient feature representation. By contrast, the bottleneck in CED-ECM-processed groove inspection is the lack of input information under small-sample, low-contrast, and single-channel imaging conditions. Simply stacking feature-enhancement modules cannot fully compensate for this physical information deficit [23]. How to explicitly inject complementary physical priors from a single grayscale image without increasing acquisition cost, while achieving lightweight and robust groove instance segmentation under small-sample and real-time constraints, remains insufficiently studied.

To address these challenges, this study proposes a pseudo-multimodal wavelet network (PMW-YOLO), a lightweight instance segmentation model for automated inspection of CED-ECM-processed shallow grooves. Instead of continuing to stack complex modules inside the network, the proposed method injects physical priors at two levels: input representation and downsampling structure. At the input end, a single grayscale image is expanded into three complementary channels. At the structural level, high-frequency boundary information is retained while model complexity is reduced. The main contributions are as follows:

First, this study expands a single-channel grayscale microscopic image into three complementary channels, namely the original grayscale image, a CLAHE-enhanced grayscale image, and multiscale Sobel gradients. This explicitly injects illumination robustness and edge priors into the network without additional acquisition hardware.

Second, this study embeds a DWTDown into the backbone network. The module uses fixed Haar wavelet decomposition to preserve LL, LH, HL, and HH subband information during early downsampling. It is treated as a lightweight structural component that maintains baseline-level segmentation accuracy with fewer parameters and lower GFLOPs, rather than as a stable source of accuracy improvement.

Finally, this study further examines the effects of EMA, boundary-aware mask loss, and data-centric augmentation. It also reports attempts that did not produce the expected benefit, such as specular-highlight hard negative mining, providing practical boundaries for similar small-sample industrial inspection tasks.

## 2. CED-ECM Process Principle and Image Detection Method

### 2.1. CED-ECM Process Principle and Image Acquisition

**Figure 1:**
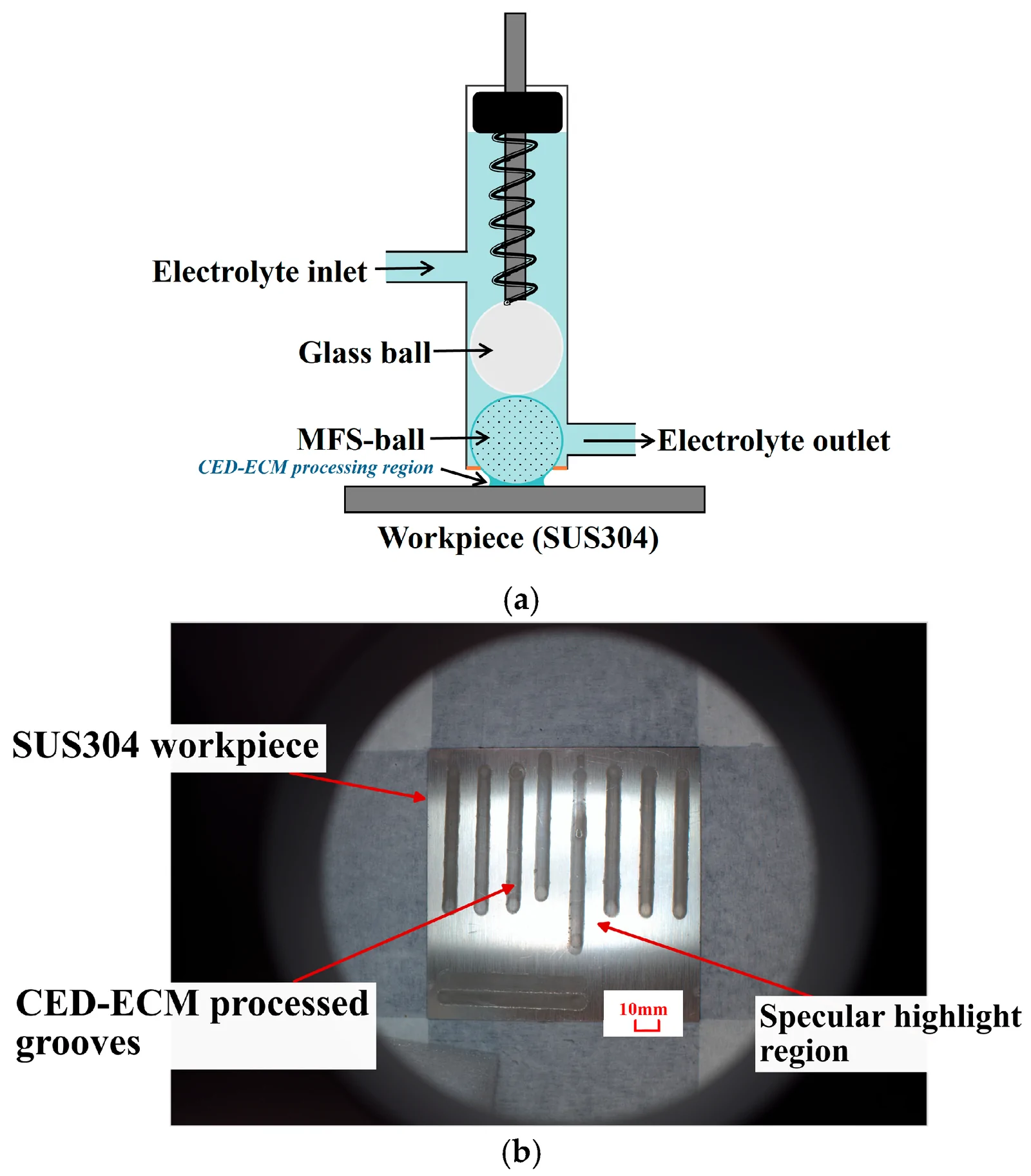
(**a**) Schematic diagram of CED-ECM. (**b**) Full-field microscopic image of a CED-ECM-processed surface, illustrating the shallow groove imaging scene and the surrounding metal–substrate texture.

CED-ECM removes material from the anodic metal surface by precisely controlling electrolyte distribution in the gap between the cathode tool and the workpiece. As shown in Figure 1a, during CED-ECM processing, a porous solid ball (MFS-ball) locally confines the electrolyte near the cathode tool, while the SUS304 workpiece serves as the anode. Under an applied voltage, localized anodic dissolution occurs within the narrow machining gap, and relative motion between the tool and the workpiece progressively forms a shallow groove while limiting stray corrosion outside the CED-ECM region. This process enables the fabrication of shallow grooves with depths of approximately several micrometers to tens of micrometers on precision metal surfaces [4,5,6,7]. The object studied in this work is the shallow groove formed on SUS304 stainless-steel surfaces by CED-ECM.

For image acquisition, the machined workpiece surfaces were captured using an optical microscopic imaging system. A full-field microscopic image of a CED-ECM-processed surface is shown in Figure 1b. In intelligent scraping-related manufacturing, the machine-vision system must identify CED-ECM-processed regions, extract workpiece contours and CED-ECM-processed grooves, and distinguish successful machining from ineffective processing because machine-driven CED-ECM tools may not produce identical outcomes in every run. A high-definition industrial camera and ring light were used to provide auxiliary illumination and improve imaging uniformity, yielding high-resolution grayscale micrographs of 5472 × 3648 pixels. Under this imaging condition, the median length of a single CED-ECM-processed groove was approximately 1021 pixels. These grooves showed weak contrast and blurred boundaries relative to the metal substrate. Some regions also contained bright specular highlights caused by surface reflection, which could be confused with groove features in both grayscale intensity and edge response.

Accordingly, the predicted instance masks provide a basis for automated groove identification and, after spatial calibration, for subsequent quantitative measurements of groove length, width, area, and boundary integrity; dimensional metrology itself is beyond the scope of the present study.

### 2.2. Dataset Construction

A total of 85 high-resolution grayscale micrographs were initially acquired. After quality screening, 83 valid original images were retained for annotation and experiments. CED-ECM-processed groove contours were annotated instance by instance as polygons using LabelMe (LabelMe 5.9.1). Because this task is a single-class CED-ECM-processed shallow groove instance segmentation problem, the number of annotated classes is nc = 1, and the foreground class corresponds to groove instances.

To prevent local tiles from the same original image from crossing the training, validation, and test sets and causing data leakage, the dataset was split at the original-image level. Using a fixed random seed of 42, the 83 valid original images were divided into 59 training images, 12 validation images, and 12 test images.

**Figure 2:**
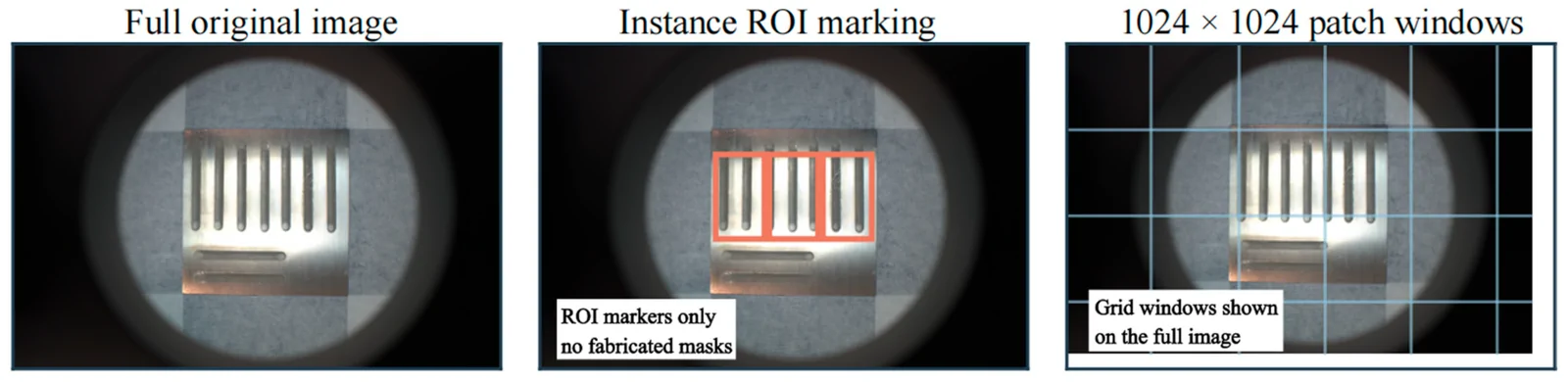
Dataset construction workflow, including the original micrograph, annotated groove regions, and the 1024 × 1024-pixel tiling window layout used for model input.

The original micrographs are high-resolution images of 5472 × 3648 pixels. Directly resizing an entire image to the model input size would compress the micrometer-scale groove width, boundary, and weak-contrast information, degrading small-object segmentation. Therefore, as shown in Figure 2, the train/validation/test split was first completed at the original-image level, and each subset was then tiled internally into 1024 × 1024-pixel local patches. These tiles are model input units cropped from the original micrographs and should not be interpreted as additional independent original samples. The entire field was retained because deployment is performed on full micrographs rather than on manually pre-cropped workpiece regions.

During training, target-containing tiles were cropped using annotated polygon centroids as anchors with random jitter, allowing the model to observe groove instances at different positions and contexts. A small number of background-only tiles, including tiles outside the workpiece region, were retained as negative samples to suppress false positives caused by support structures, specular highlights, and surrounding texture; these tiles were not treated as groove instances. Nevertheless, because all images were acquired using one setup, learning of background-specific cues cannot be completely excluded. During validation and testing, each original image was tiled using non-overlapping regular-grid sliding windows, and both target-containing and background-only windows were retained. This protocol simulates full-image deployment and evaluates both missed detections and false positives.

The final number of tiles was 423 for training, including 40 background tiles; 288 for validation, including 203 background tiles; and 288 for testing, including 234 background tiles. All tiles inherited the data split of their parent original image, so no tile from the same original image crossed training, validation, and test subsets.

### 2.3. Pseudo-Multimodal Channel Fusion

Grayscale microscopic imaging provides only a single intensity channel, and the weak contrast and blurred boundaries of CED-ECM-processed grooves make single-channel input insufficient for feature representation. Inspired by multimodal and multi-view feature fusion [24], this study constructs three physically complementary channels from a single grayscale image without additional acquisition hardware, explicitly injecting illumination robustness and edge priors into the network input.

Given a single-channel grayscale image, the fused output is a three-channel tensor:

Channel 1: The original grayscale image, which preserves the original texture and intensity information as the basic feature representation.

Channel 2: The CLAHE-enhanced grayscale image. Contrast Limited Adaptive Histogram Equalization (CLAHE) [25] is applied with clip_limit = 2.0 and tile = 8 × 8 to enhance local contrast and highlight weak grooves in low-contrast regions.

Channel 3: Multiscale Sobel gradients. Sobel gradient magnitudes sqrt(*Gx*^2^ + *Gy*^2^) are computed and fused at kernel sizes *k* = 3, 5, and 7 to strengthen boundary responses for grooves of different widths.

The multiscale gradient in Channel 3 is constructed according to Equations (1) and (2). For the grayscale image, horizontal and vertical Sobel gradients are calculated at different scales. Their magnitudes are then averaged across the three scales:

(1) $$M=\frac{1}{|K|}\sum_{k\in K}\sqrt{{\left(G_{x}^{\left(k\right)}\right)}^{2}+{\left(G_{y}^{\left(k\right)}\right)}^{2}},K=\{3,5,7\}$$

The averaged multiscale Sobel magnitude *M* is independently min–max normalized within each tile to obtain the third channel *C*_3_. As implemented in the code, a tile with a near-constant response is mapped to zero; otherwise, min–max normalization is applied:

(2) $$C_{3}=255\times \frac{M-min(M)}{\max\left(M\right)-\min\left(M\right)+\varepsilon }$$

The final pseudo-multimodal input is obtained by stacking the three channels, as shown in Equation (3):

(3) $$X=stack\left[I,\;CLAHE(I),\;C_{3}\;\right]\in R^{1024\times 1024\times 3}$$

**Figure 3:**
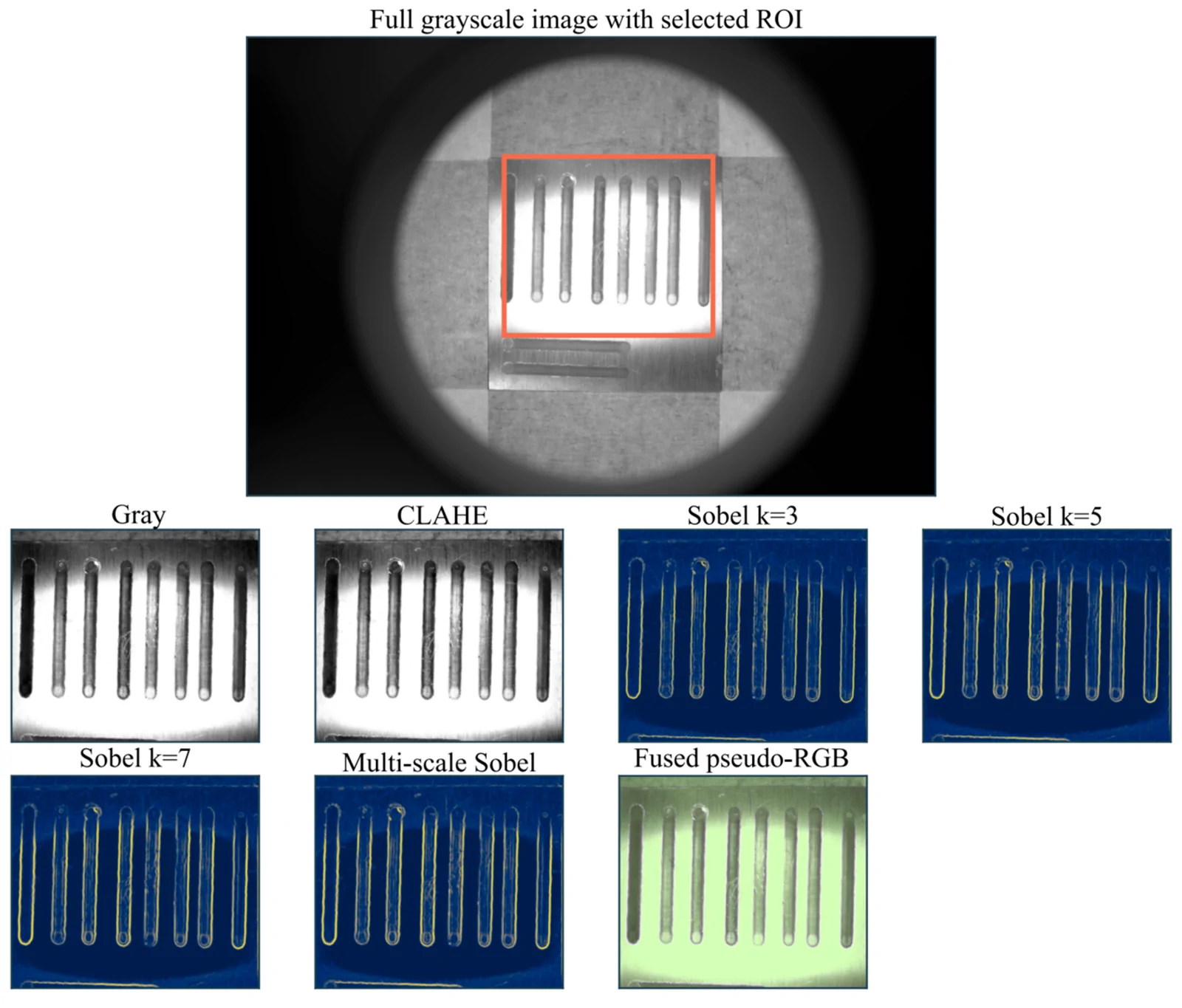
Pseudo-multimodal channel fusion workflow. A single-channel grayscale micrograph is transformed into three complementary channels: original grayscale, CLAHE-enhanced grayscale, and multiscale Sobel gradient.

The three channels correspond to the original grayscale image, the CLAHE-enhanced grayscale image, and the multiscale Sobel gradient. Small kernels respond to narrow grooves, large kernels respond to broad and gradual boundaries, and multiscale averaging accommodates grooves with different widths. The pseudo-multimodal channel fusion workflow is shown in Figure 3.

This design has three advantages. The grayscale channel preserves texture, the CLAHE channel enhances local contrast, and the Sobel channel emphasizes boundaries. The same deterministic preprocessing is applied before both training and inference, without requiring additional sensing hardware. Because all three channels are derived from the same grayscale image, the design should be interpreted as grayscale-derived feature-channel engineering rather than true multimodal sensing or learned cross-modal fusion. The gain of the three-channel representation over conventional RGB input is quantitatively evaluated through controlled ablations in Section 3.

### 2.4. PMW-YOLO Network Architecture

**Figure 4:**
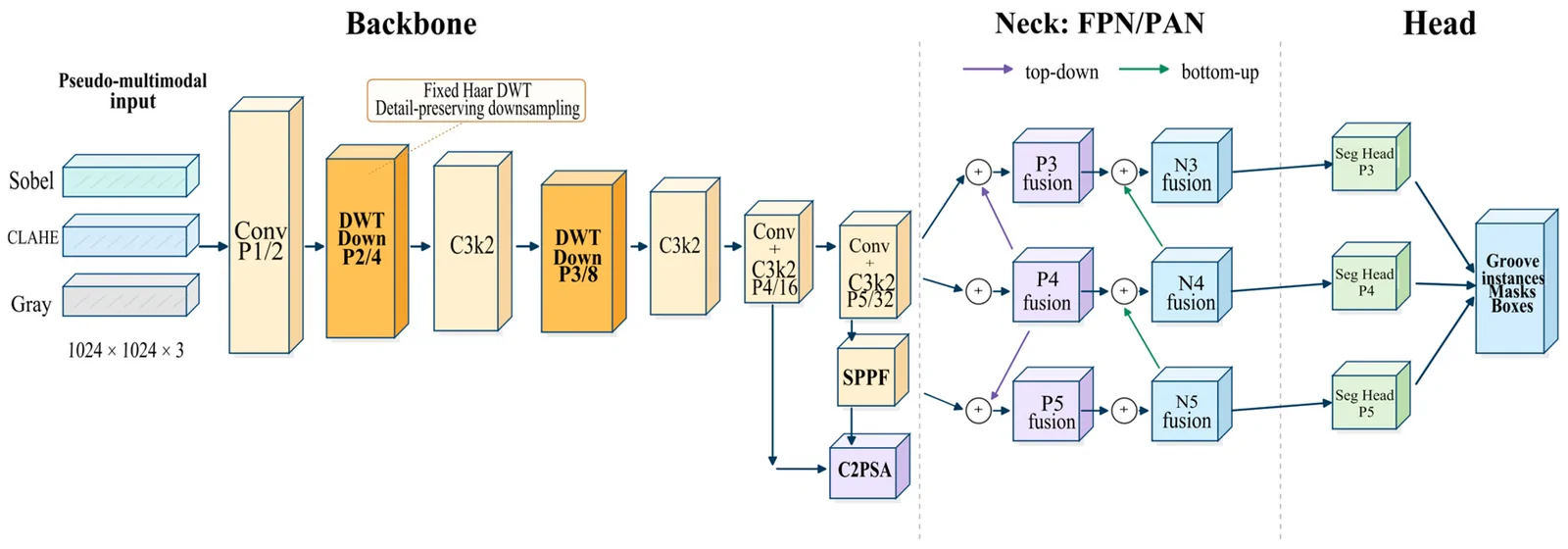
PMW-YOLO network structure. DWTDown is embedded in the early P2/4 and P3/8 downsampling stages of the backbone, followed by FPN/PAN neck fusion and Segment-head output of bounding boxes and instance masks.

PMW-YOLO uses the lightweight n-scale segmentation model yolo26n-seg from YOLO26-seg as the baseline framework. As shown in Figure 4, this model balances speed and accuracy in a one-stage instance segmentation framework, and its backbone, neck, and detection head are suitable for module-level modification. With the pseudo-multimodal input, the baseline model has 2,689,079 parameters and 28.46 GFLOPs. The computational cost was measured using the PyTorch profiler (PyTorch 2.8.0) at an input size of 1024 in the unfused setting.

**Figure 5:**
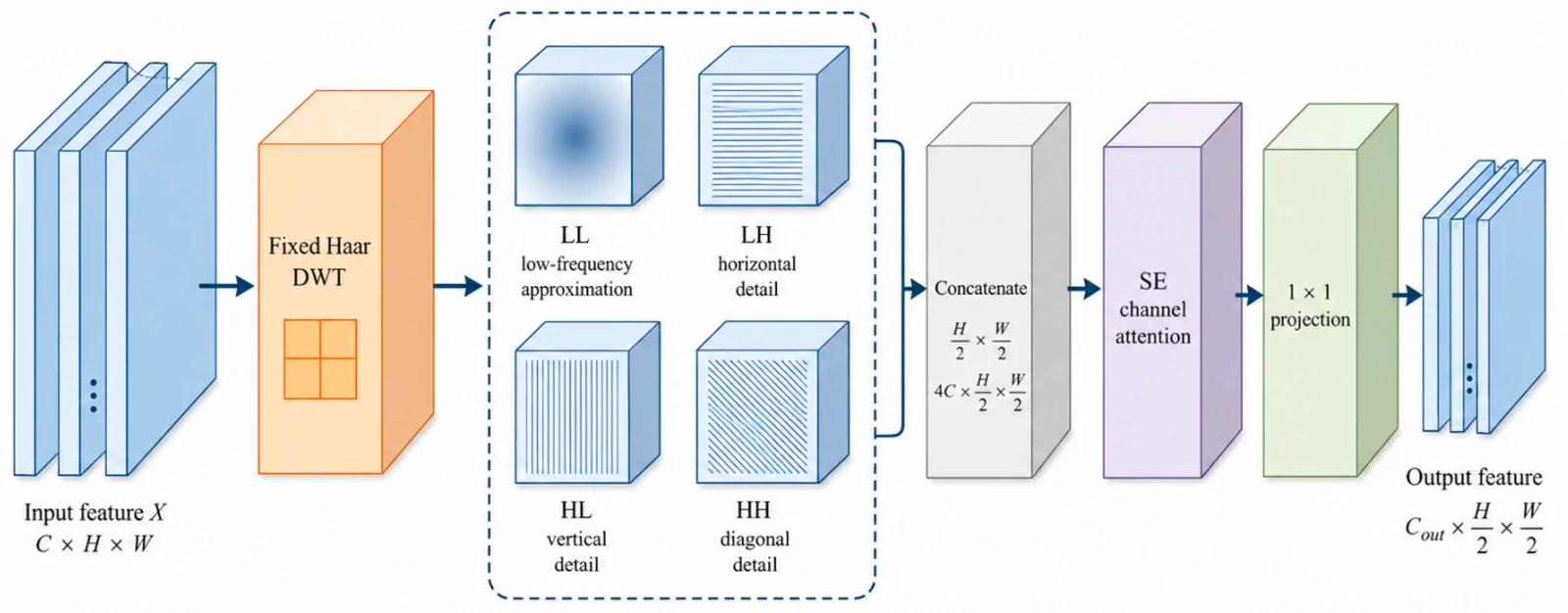
Structure of the DWTDown module. Input features are first decomposed by fixed Haar wavelets into the LL low-frequency approximation subband and the LH, HL, and HH high-frequency subbands, and are then concatenated, reweighted by SE channel attention, and projected by a 1 × 1 convolution.

Conventional stride convolution downsampling reduces spatial resolution but can lose high-frequency information, whereas groove boundaries are concentrated in high-frequency components. Therefore, this study designs a DWTDown to replace part of the stride-2 convolution layers in the backbone. The structure of the DWTDown module is shown in Figure 5. The module uses fixed Haar wavelet bases to decompose the input into a low-frequency approximation component and three high-frequency subbands in the horizontal, vertical, and diagonal directions. It then applies lightweight SE channel attention to the 4 × c1 subbands and projects them to the target channel number through a 1 × 1 convolution.

Specifically, for each input channel, four 2 × 2 Haar kernels are applied as stride-2 grouped convolutions, producing the LL low-frequency approximation subband and the LH, HL, and HH high-frequency subbands, as shown in Equation (4):

(4) $$h_{LL}=\frac{1}{2}\left[\begin{array}{cc} 1 & 1\\ 1 & 1 \end{array}\right],h_{LH}=\frac{1}{2}\left[\begin{array}{cc} 1 & 1\\ -1 & -1 \end{array}\right],\;h_{HL}=\frac{1}{2}\left[\begin{array}{cc} 1 & -1\\ 1 & -1 \end{array}\right],\;h_{HH}=\frac{1}{2}\left[\begin{array}{cc} 1 & -1\\ -1 & 1 \end{array}\right]$$

The subband tensor obtained from grouped convolution is then reweighted by SE channel attention and projected to the target channels through a 1 × 1 convolution, as shown in Equation (5):

(5) $$F^{\prime }=\phi \;\left(\;F_{\mathrm{sub}}⊙A(F_{\mathrm{sub}})\;\right),F^{\prime }\in R^{C^{\prime }\times \frac{H}{2}\times \frac{W}{2}}$$

Here, channel-wise multiplication denotes the reweighting of subband features by SE weights. The squeeze-and-excitation structure consists of global average pooling, 1 × 1 channel reduction, SiLU, 1 × 1 channel expansion, and Sigmoid. The Haar kernels are fixed and introduce no learnable weights. Because the Haar filters are fixed buffers, the module reduces the learnable parameters introduced by early downsampling. In PMW-YOLO, DWTDown is deployed at the P2/4 and P3/8 downsampling positions of the backbone.

The PMW-YOLO backbone sequentially contains Conv-P1/2, DWTDown-P2/4, C3k2, DWTDown-P3/8, C3k2, Conv-P4/16, C3k2, Conv-P5/32, C3k2, SPPF, and C2PSA. The neck adopts bidirectional feature fusion through FPN and PAN, and the detection head is the Segment segmentation head.

### 2.5. Boundary-Aware Mask Loss

**Figure 6:**
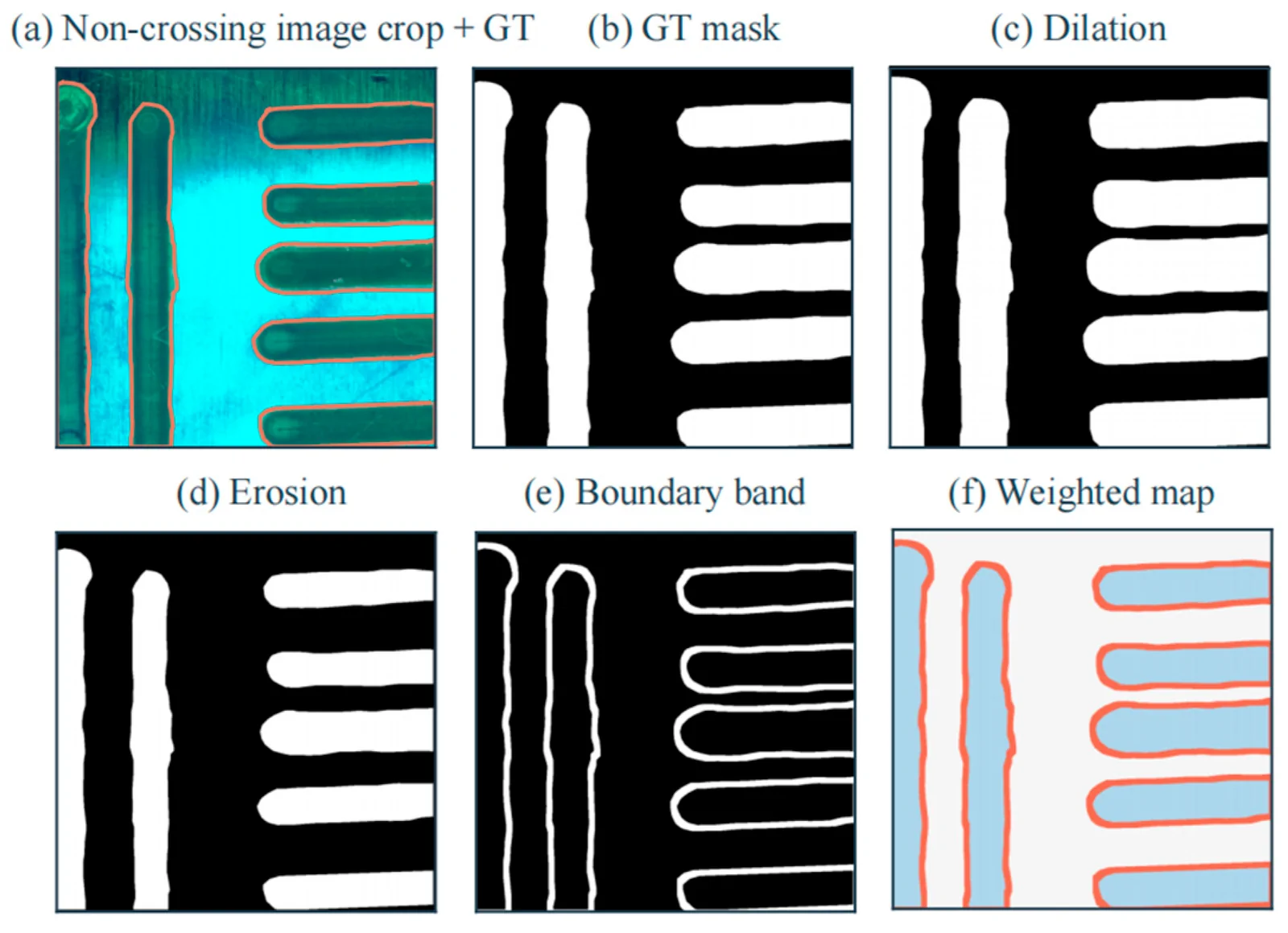
Calculation workflow of the boundary-aware mask loss. (**a**) Image crop and ground-truth annotation; (**b**) ground-truth mask; (**c**) dilation result; (**d**) erosion result; (**e**) boundary band obtained from the difference between dilation and erosion; (**f**) boundary-weight map used for the additional loss.

To address blurred groove boundaries and insufficient instance-boundary learning for irregular shapes, this study introduces a boundary-aware mask loss, BoundaryMaskLoss. The calculation workflow of the boundary-aware mask loss is shown in Figure 6. The loss extracts a boundary band from the ground-truth mask using a morphological gradient, approximated as the difference between dilation and erosion implemented by max pooling. BCE and Dice/Tversky-type overlap losses [26] are then computed only within this boundary band, encouraging the model to focus on accurate boundary segmentation.

Specifically, for a binary ground-truth mask, dilation and erosion are implemented using max pooling with the specified kernel size, and their difference defines the boundary band, as shown in Equation (6):

(6) $$B=clip(D(G)-E(G),\;0,\;1),E(G)=-\;{MaxPool}_{k}(-G)$$

Given the predicted mask logits as input, the boundary loss accumulates BCE and soft-Dice only over the boundary band, as shown in Equation (7):

(7) $$L_{\mathrm{boundary}}\;=\;w_{b}\text{⋅}\frac{\text{∑}(B\text{⊙}\mathrm{BCE}(\hat{P},G))}{\text{∑}B}\;+\;w_{d}\left(1|\frac{2\text{∑}(\sigma (\hat{P})\;\text{⊙}\;B)(G\text{⊙}B)+\varepsilon }{\text{∑}(\sigma (\hat{P})\text{⊙}B)+\text{∑}(G\text{⊙}B)+\varepsilon }\right)$$

Here, Sigmoid converts logits into probabilities, and the smoothing term prevents division by zero. This loss is a zero-parameter design with no learnable weights and is compatible with distributed training. During training, it is added as a weighted term to the original segmentation loss, as shown in Equation (8):

(8) $$L_{\mathrm{total}\;}={\;L}_{\mathrm{seg}}\;+{\;\lambda }_{\mathrm{edge}}\text{⋅}L_{\mathrm{boundary}}$$

The boundary-aware term is evaluated only within the extracted boundary band. However, it is added to the original segmentation loss L_seg, which continues to supervise the complete groove mask. Thus, the auxiliary term strengthens contour localization, whereas the network can still learn intensity, texture, shape, and contextual cues within and around the groove through the original full-mask objective. The boundary gain is controlled by the hyperparameter edge. The default value is edge = 0.0, which is identical to baseline training behavior, and edge = 0.5 is used in the experiments. In this study, the boundary loss is used as an ablation item to examine the effect of boundary constraints. Three-fold cross-validation repeated-holdout analysis shows that its gain in Mask mAP50-95 is smaller than the variation across runs. It is therefore not presented as an independent core contribution. The related results are mainly used to show that boundary constraints have a positive trend in recall, which still requires further validation on larger datasets.

### 2.6. Data-Centric Augmentation Strategy

After baseline performance reached a usable level, the remaining engineering errors were false positives from specular highlights and missed detections of irregular grooves. Because the bottleneck of this task lies in the small-sample condition of 83 valid original images rather than merely insufficient network representation, this study does not continue to stack network modules. Instead, it adopts a data-centric strategy and designs two targeted augmentations for comparison:

Copy-Paste instance augmentation is used to reduce missed detections. In baseline training, copy_paste = 0.0. When copy_paste = 0.3 is enabled, real groove instances, especially irregular instances, are pasted onto different backgrounds to increase morphological diversity at low cost [27].

Specular-highlight hard negative mining is used to reduce false positives. Background windows originally sampled at random are replaced by windows preferentially collected from overexposed or bright regions without annotations. Following the idea of online hard example mining [28], this explicitly trains the model to distinguish highlights from grooves. A sampling check showed that this strategy collected more specular-highlight regions. The mean brightness of background tiles increased from 44.7 to 70.4, and the saturated-pixel ratio increased from 0.0047 to 0.0106.

The actual effects of the two strategies, including attempts that did not yield the expected benefit, are quantitatively compared in Section 3.

### 2.7. Training Settings and Evaluation Metrics

All experiments were conducted under a unified configuration: an input tile size of 1024 × 1024 pixels, 500 training epochs, a batch size of 16, mixed precision enabled, and a fixed random seed of 42. The optimizer and learning-rate schedule followed the default Ultralytics settings. The model was implemented based on Ultralytics 8.4.58, and training and inference were performed on a single NVIDIA RTX 5070 Ti GPU.

The native Ultralytics mAP evaluation system was used. For the single CED-ECM groove class, Box and Mask mAP@0.5, mAP@0.5:0.95, precision P, and recall R were reported. Unless otherwise stated, mAP, P, and R are reported as fractions between 0 and 1 rather than percentages. Deployment-related metrics, including parameter count, GFLOPs, and inference speed, were also reported. All primary model comparisons were evaluated on the held-out test set after the training settings had been fixed.

**Figure 7:**
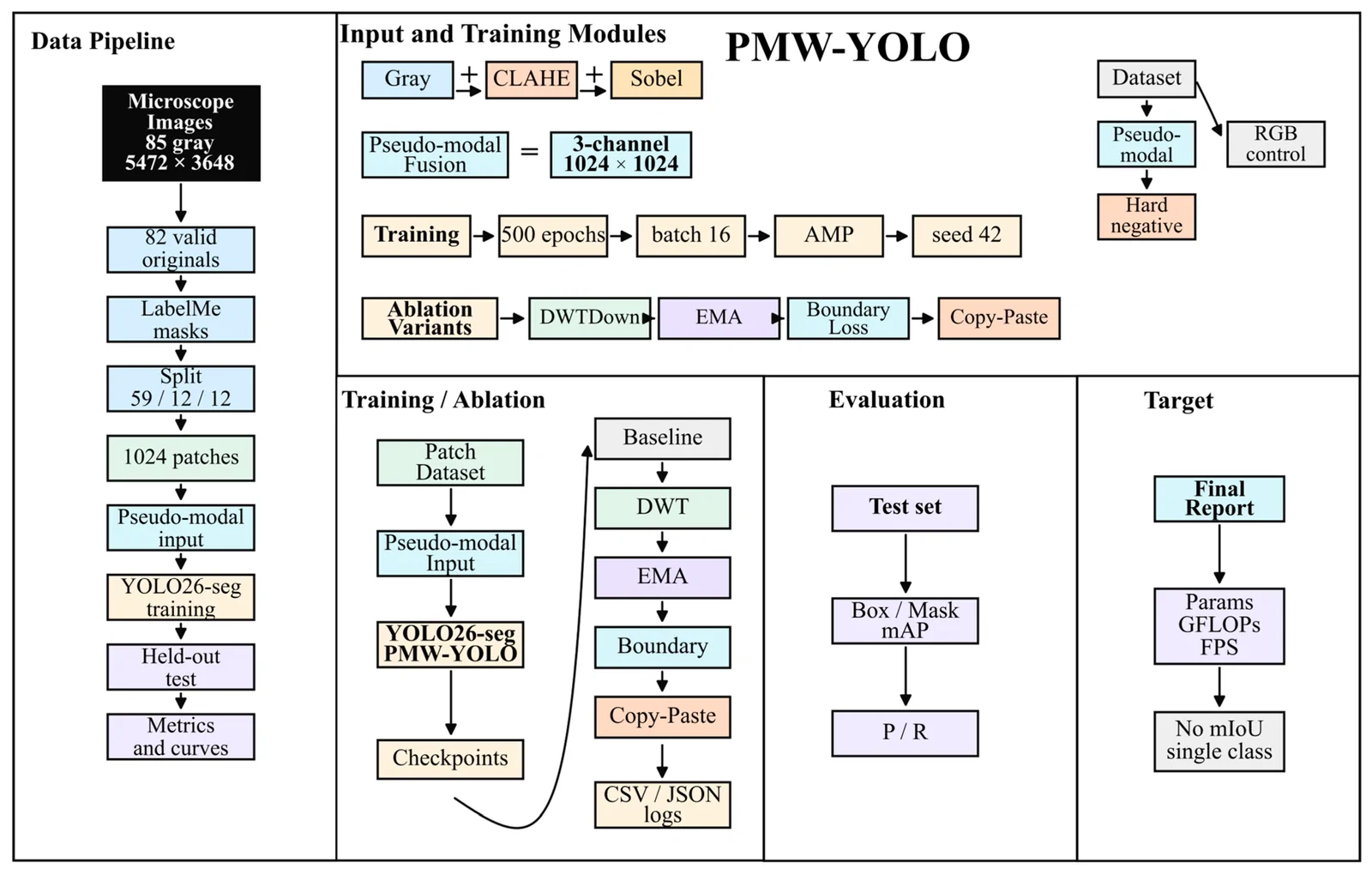
Controlled experimental workflow, including input construction, model training, inference evaluation, error analysis, and ablation experiments.

Because this task is single-class instance segmentation and the foreground corresponds to grooves, semantic segmentation mIoU was not used. The controlled experimental workflow, including the input construction, model training, inference evaluation, error analysis, and ablation experiments, is shown in Figure 7.

## 3. Results and Discussion

### 3.1. Ablation Experiments on Architecture and Loss

**Figure 8:**
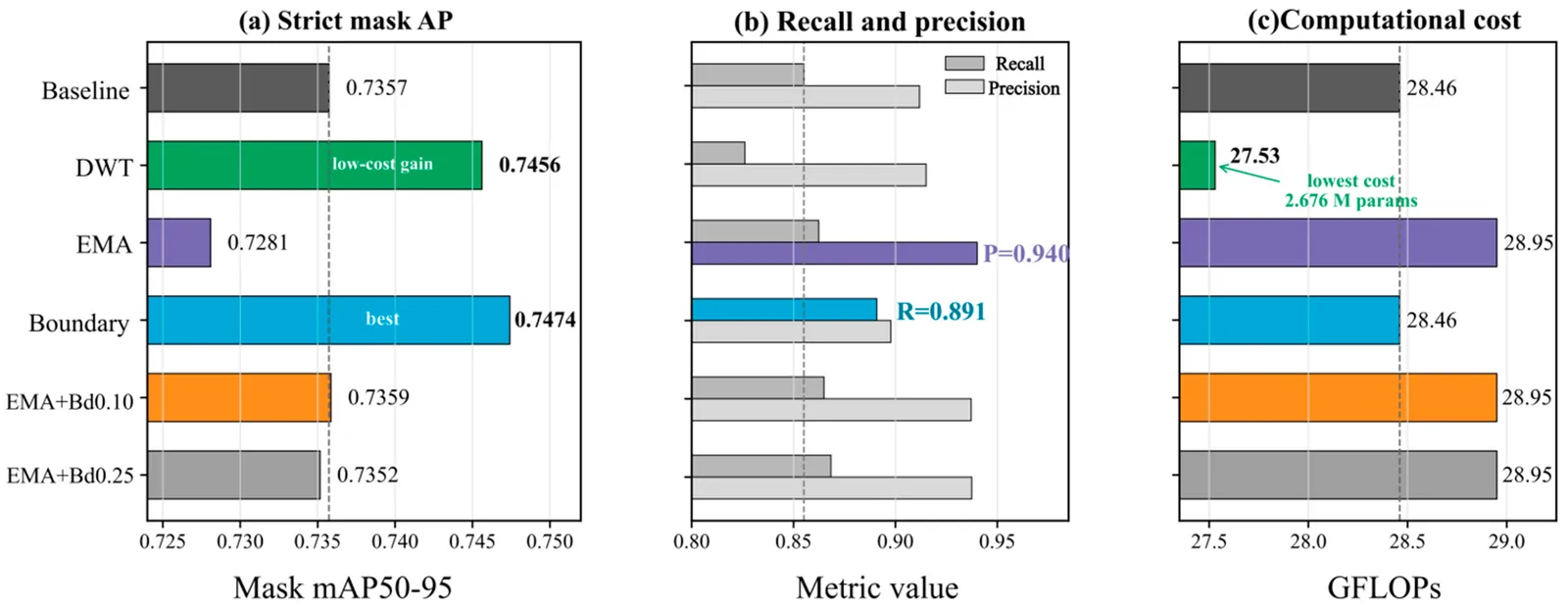
Single-split architecture and loss ablation results. (**a**) Strict Mask mAP50-95 comparison among variants; (**b**) precision and recall comparison; (**c**) computational cost measured by GFLOPs.

To clarify the contribution of each improvement, DWTDown, EMA, and boundary-aware loss were individually and jointly added to a unified baseline consisting of YOLO26-seg with pseudo-multimodal three-channel input. Their individual and combined effects were examined. The test set results are shown in Table 1. The single-split architecture and loss ablation results are shown in Figure 8.

Bold indicates the best value in each column. GFLOPs were measured by torch profiler at input size = 1024 using the unfused setting.

After DWTDown was added, the parameter count decreased to 2,675,599, the lowest value in the table and about 13,500 fewer than the baseline. GFLOPs decreased to 27.53, also the lowest value. At the same time, Mask mAP50-95 reached 0.7456, slightly higher than the baseline value of 0.7357, while Box mAP50-95 remained comparable to the baseline. This indicates that DWTDown can achieve segmentation accuracy comparable to the baseline with lower parameter and computational costs. This lightweight property is determined by the module structure, does not depend on a specific random seed, and may benefit online inspection scenarios requiring edge deployment.

Without adding parameters, the boundary-aware loss achieved the highest Mask mAP50, Mask mAP50-95, and recall R in this table, indicating that boundary constraints may help reduce missed detections.

EMA achieved the highest precision P (0.940), but Box/Mask mAP50-95 decreased slightly, showing an overall pattern of high precision and relatively conservative detection.

The variant combining all three improvements achieved a Mask mAP50-95 of 0.7373, lower than the best single improvement. The combination of EMA and boundary loss also did not outperform boundary loss alone. This suggests that, under the small-sample condition of this task, simple combinations of improvements do not lead to linearly accumulated gains.

### 3.2. Repeated Original-Image-Level Holdout and Stability Analysis

**Figure 9:**
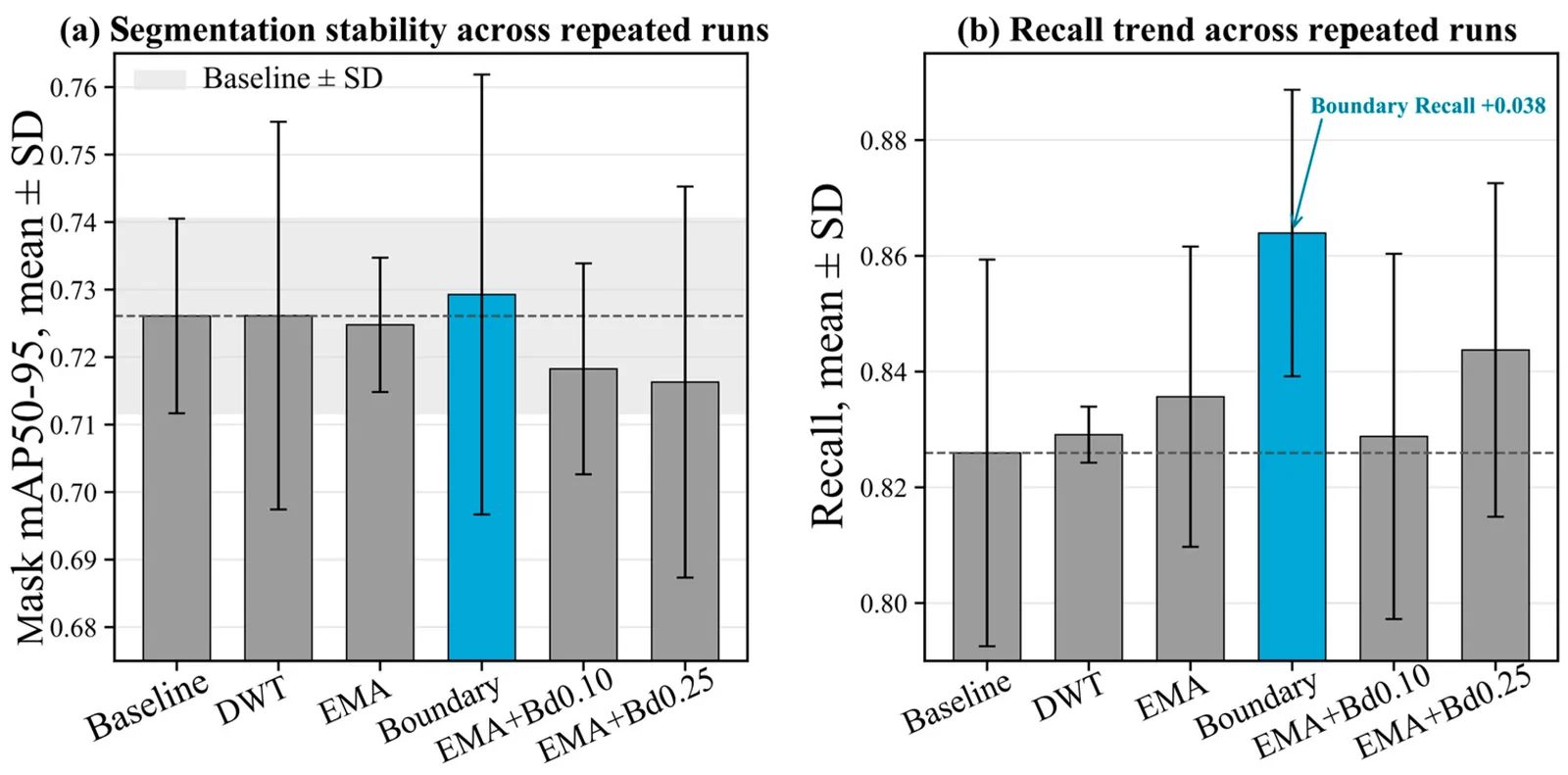
Stability analysis across three repeated original-image-level holdout runs. (**a**) Mean and standard deviation of Mask mAP50-95; (**b**) mean and standard deviation of recall under small-sample conditions.

Because the dataset is small, metrics obtained from a single split may be affected by data partition and training randomness. To assess the robustness of the conclusions, this study repeated training and evaluation using three original-image-level holdout splits generated with seeds 42, 1, and 2, with the corresponding seeds also used for model training. Each run used 59 training, 12 validation, and 12 test images, and all variants were trained for 500 epochs. Because the three splits overlap and are not mutually exclusive folds, the mean and standard deviation of Mask mAP50–95 and recall quantify sensitivity to internal splitting and training randomness rather than three-fold cross-validation. The results are shown in Table 2 and Figure 9.

The repeated-holdout results show that, for Mask mAP50-95, the means of all improved variants fall within one standard deviation of the baseline. Their gains over the baseline are therefore insufficient to support a conclusion of stable improvement. For example, the boundary-loss variant has the highest mean value, 0.7293, but its standard deviation is 0.0326, much larger than the mean gain of 0.0032 over the baseline. The DWT mean equals the baseline mean, both at 0.7261. Rankings also fluctuate across runs, indicating that the best result from a single split should be interpreted cautiously together with repeated-run results.

Based on this observation, the boundaries of the conclusions for each improvement are defined as follows:

The lightweight nature of DWTDown is a structural fact and does not depend on the random seed. The parameter count of 2.676 M and the computational cost of 27.53 GFLOPs are determined by the module structure, while its segmentation accuracy is comparable to the baseline across three runs. Therefore, the reliable conclusion for DWTDown is that it achieves comparable accuracy with lower parameter and computational costs, rather than significantly improving accuracy.

At the recall level, boundary loss reaches 0.864 ± 0.025, higher than the baseline value of 0.826 ± 0.033. This shows a consistent positive trend and is consistent with its design motivation of focusing on boundaries and reducing missed detections, but it still requires further confirmation on larger datasets.

Therefore, mAP improvement from architectural modifications is not treated as the core claim of this paper. The corresponding modules are reported as ablation items. Although original-image-level splitting prevents tile-level leakage, all 83 valid micrographs were obtained from a single acquisition source. The present results should therefore be interpreted as internal validation under the represented SUS304 material, machining, illumination, and imaging conditions; generalization across machining parameters, materials, batches, backgrounds, and imaging systems remains to be evaluated.

### 3.3. Effectiveness of Pseudo-Multimodal Fusion

To verify the gain of pseudo-multimodal three-channel fusion over conventional input, this study constructed a control experiment by retraining on the RGB control dataset. The control results were compared with the corresponding pseudo-multimodal rows from Section 3.1, namely the baseline and final bd_cp models, so that the metric-wise gains of pseudo-multimodal input over RGB input can be directly evaluated. The results are shown in Table 3. The pseudo-multimodal baseline row is taken from Table 4, and the pseudo-multimodal bd_cp row is taken from the final model in Table 4.

**Figure 10:**
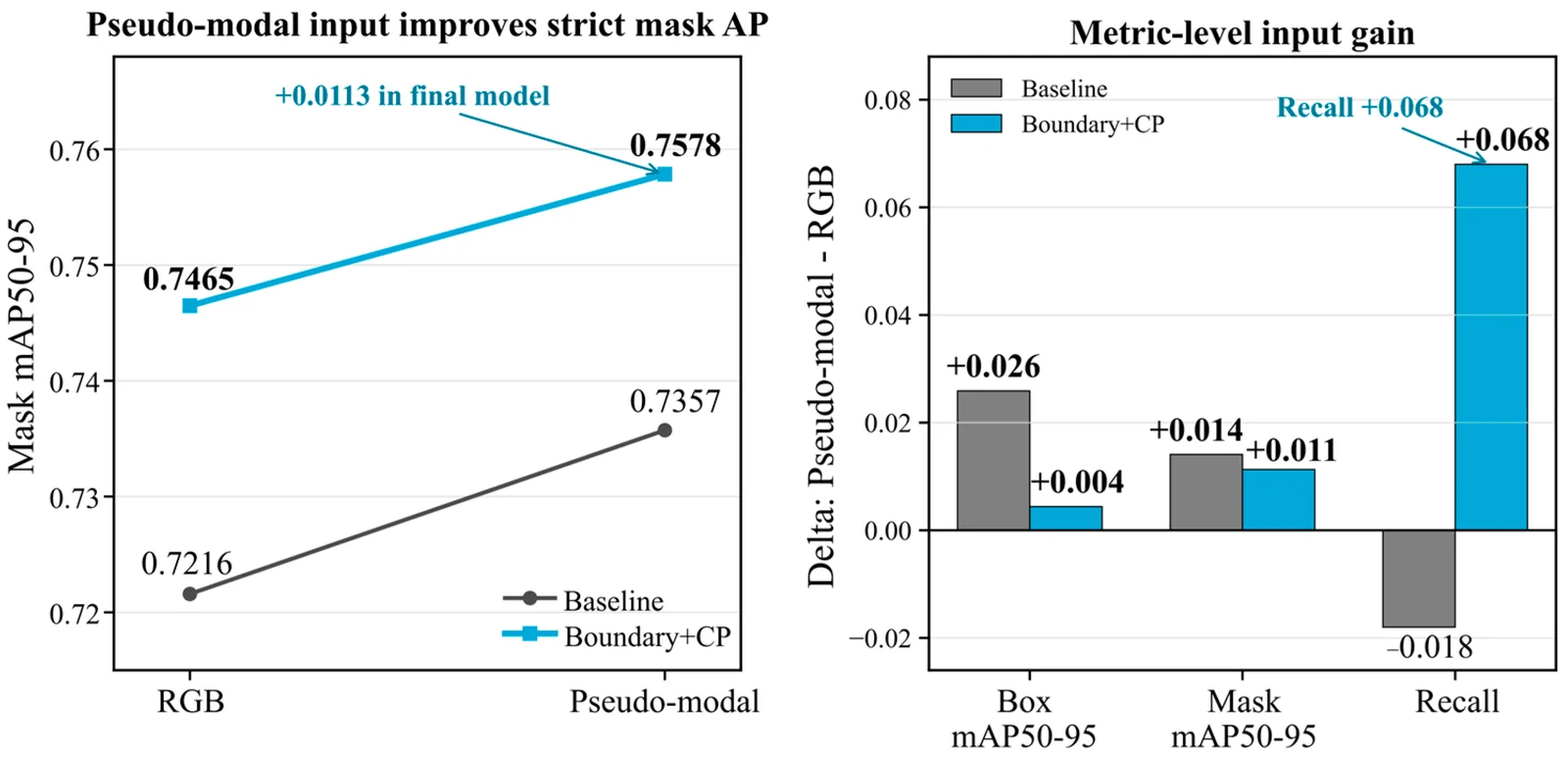
Quantitative comparison between pseudo-multimodal input and RGB control input. The pseudo-multimodal representation mainly improved strict localization metrics and recall in the boundary-loss plus Copy–Paste configuration, while not improving every metric in the baseline setting.

As shown in Table 3 and Figure 10, the control results show that pseudo-multimodal fusion provides consistent positive gains for both models on the strictest Box/Mask mAP50-95 metrics, and increases recall in the final bd_cp model from 0.831 to 0.899. This is consistent with the design motivation of injecting edge priors at the input end and improving the detectability of weak grooves. It should be noted that pseudo-multimodal input does not improve every metric, such as Mask mAP50, indicating that its benefit mainly appears under stricter localization thresholds and in recall rather than across all metrics. Overall, pseudo-multimodal fusion is an effective input-level improvement over conventional RGB input and does not require additional acquisition cost.

### 3.4. Data-Centric Augmentation Ablation

**Figure 11:**
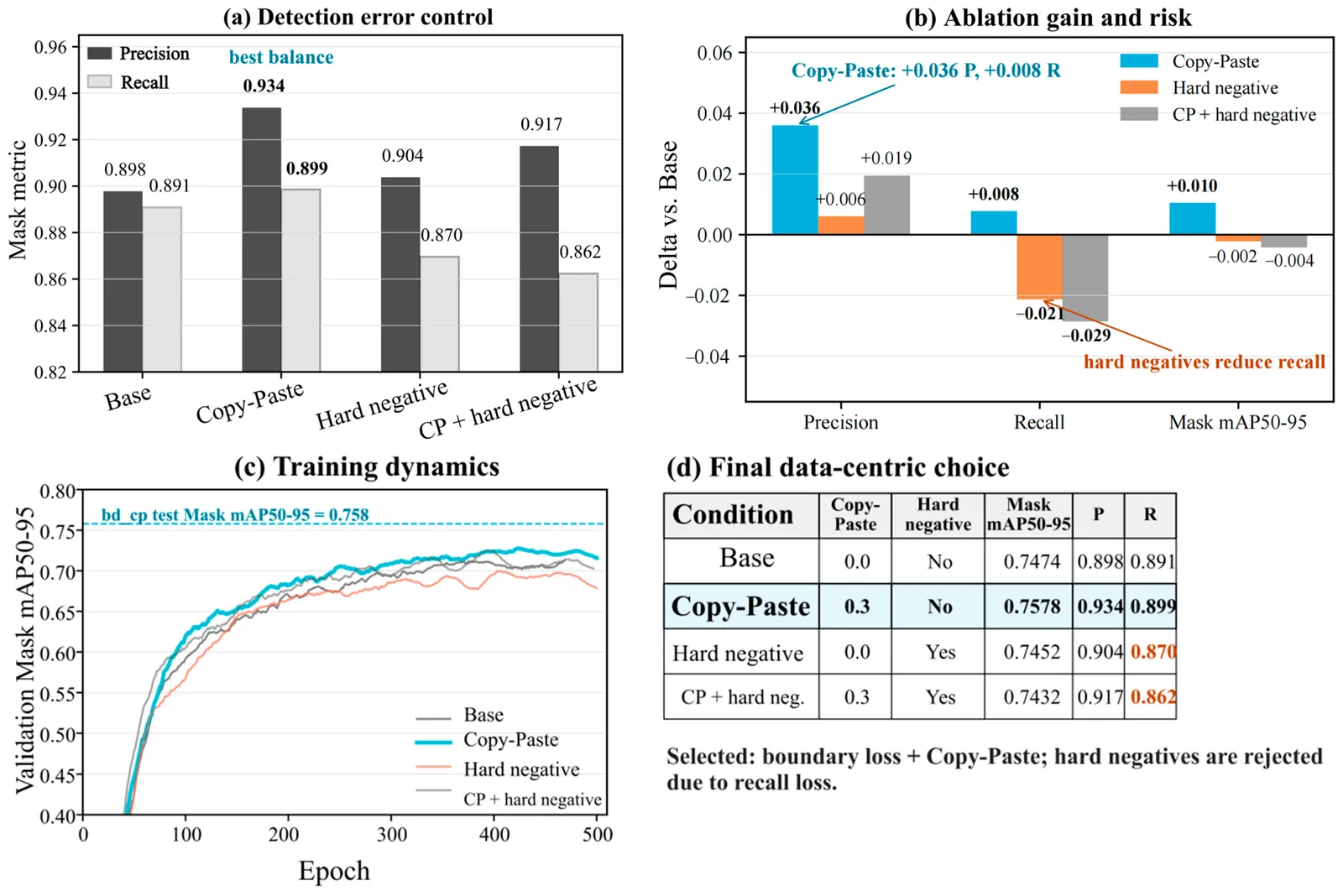
Ablation results for data-centric augmentation. (**a**) Precision–recall comparison of different augmentation settings. (**b**) Metric changes relative to the base setting. (**c**) Validation Mask mAP50-95 training dynamics over 500 epochs. (**d**) Final data-centric model selection and test metrics, showing that Copy–Paste provides the best overall trade-off among the tested strategies.

With the boundary-loss variant fixed, this study further compared Copy–Paste augmentation and hard-negative mining as post hoc data-centric analyses. These experiments were used to characterize error trade-offs under the same test protocol, rather than to perform independent validation-based model selection. The results are shown in Table 4. Ablation results for data-centric augmentation are shown in Figure 11.

An increase in P corresponds to fewer specular-highlight false positives, and an increase in R corresponds to fewer missed detections.

After Copy–Paste was enabled, precision increased from 0.898 to 0.934, recall increased from 0.891 to 0.899, and Box/Mask mAP50-95 also reached their highest values, 0.7305 and 0.7578, respectively. This gain requires only one training switch and adds no model parameters. It is consistent with previous findings on Copy–Paste instance augmentation [27] and the effectiveness of data augmentation for small-sample industrial inspection [29].

After hard negatives were introduced, recall decreased to 0.870 for bd_hn and 0.862 for bd_cphn. A possible reason is that, after many bright windows were labeled as negative samples, the model became overly conservative for true grooves that are also relatively bright, thereby increasing missed detections. Under the small-sample condition of 83 valid original images, this over-suppression outweighed the benefit of reducing false positives. This result indicates that manually constructing negative classes in small-sample scenarios requires particular caution. Natural instance augmentation through Copy–Paste is preferable to artificial negative-class injection. Under this fixed experimental setting, Copy–Paste showed the most favorable trade-off among the tested data-centric strategies, whereas hard-negative mining increased missed detections. Therefore, Copy–Paste is reported as the best-performing tested configuration in this post hoc comparison, rather than as a model selected through an independent validation procedure.

### 3.5. Qualitative Results

**Figure 12:**
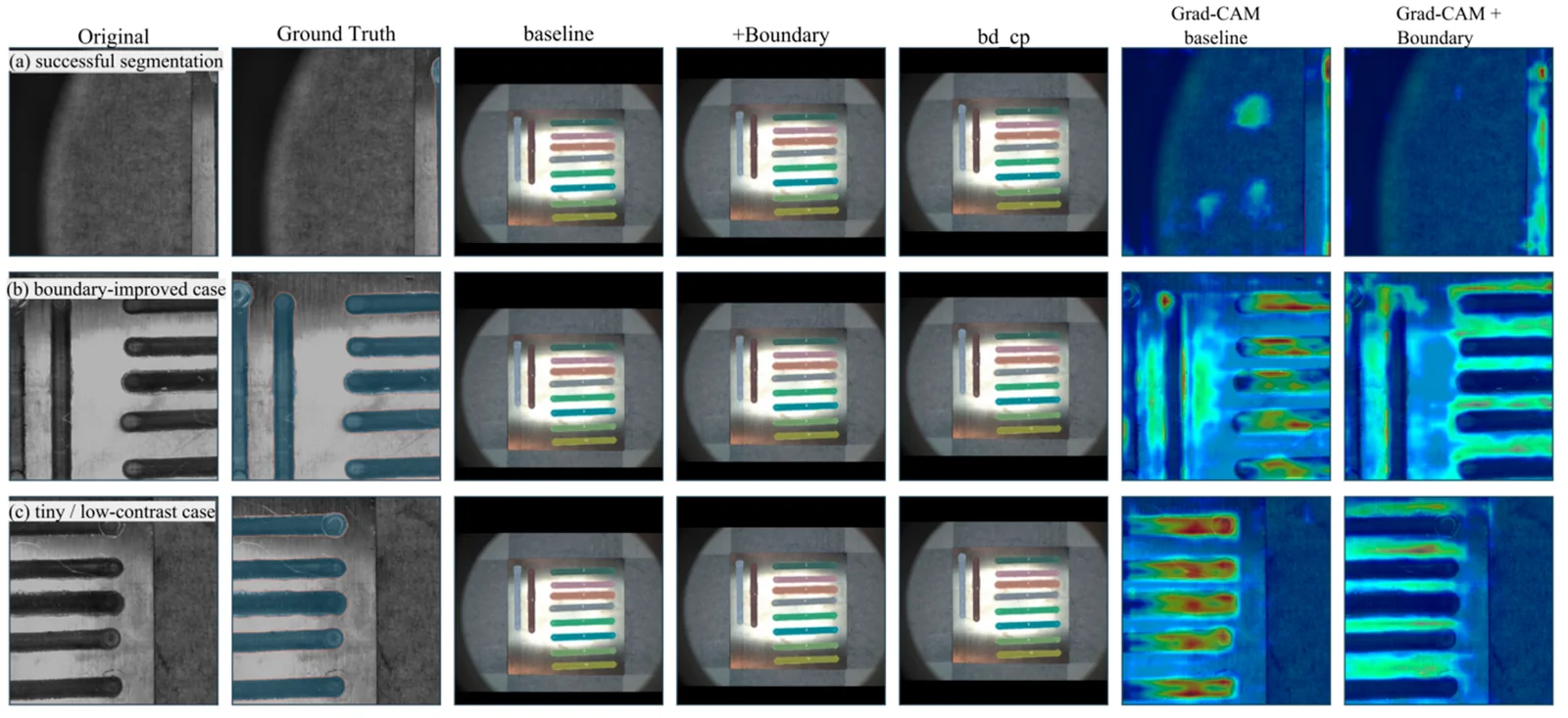
Qualitative segmentation and Grad-CAM comparison between the baseline and boundary-loss variants. The rows show representative successful, boundary-improved, and low-contrast cases; the columns compare original images, ground truth, model predictions, and activation maps.

This study further compares activation maps and instance-segmentation outputs to examine model behavior visually. As shown in Figure 12, Grad-CAM heatmaps were generated for the baseline and boundary-loss variants. Their activation distributions over groove regions were compared to qualitatively illustrate the effect of boundary-aware loss on boundary attention.

**Figure 13:**
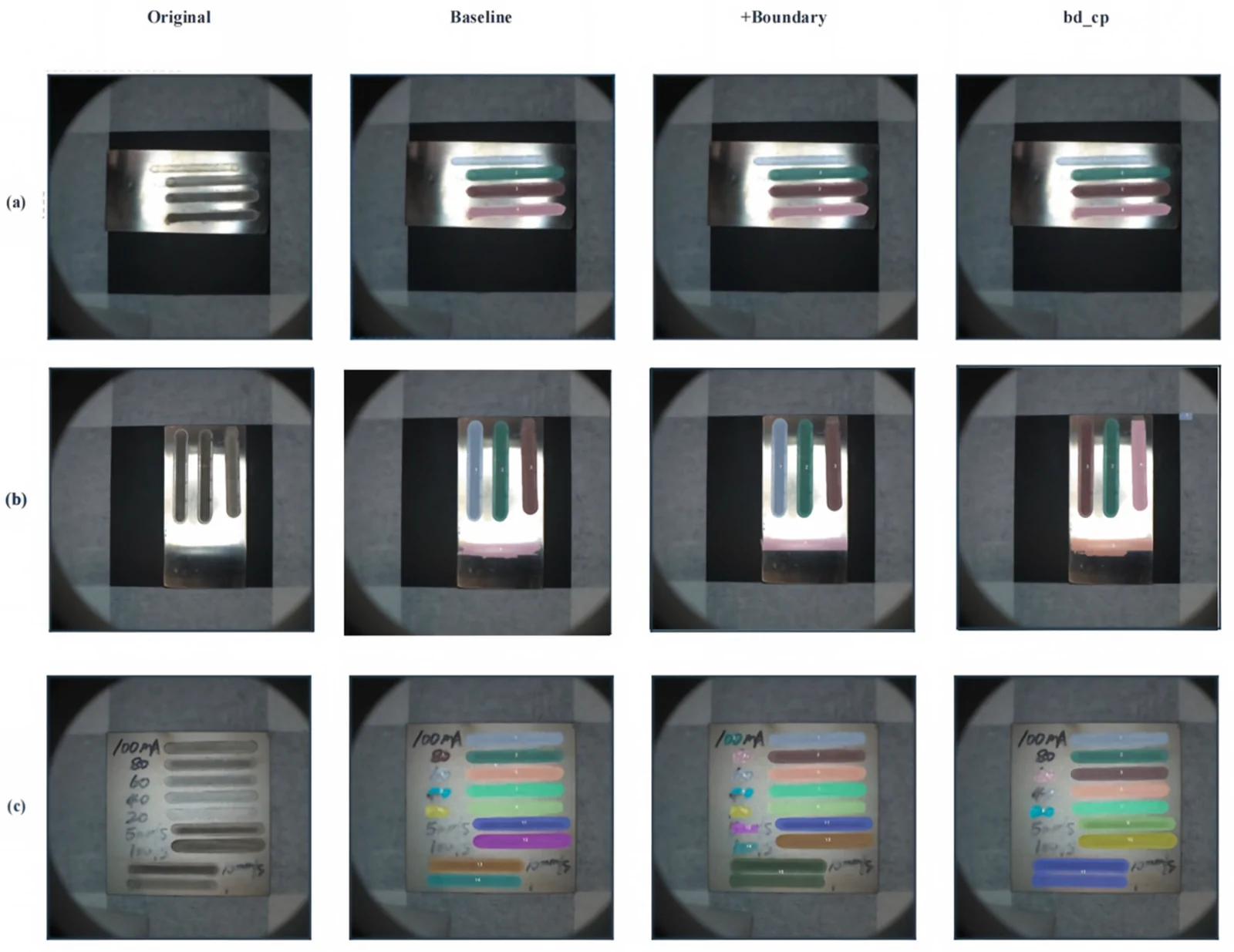
Visual comparison of instance-segmentation predictions from the Baseline, +Boundary, and bd_cp models. (**a**) Successful segmentation case comparing the original image and predictions from the three models; (**b**) boundary-improved case showing the effect of the +Boundary model on groove segmentation; (**c**) failure case showing false-positive predictions on handwritten text. Colors distinguish connected predicted regions and do not represent different semantic classes.

Instance-segmentation predictions from the three models were compared on the same full-field test images in Figure 13. The colored regions are model predictions, and different colors only distinguish connected predicted regions rather than semantic classes or manual markings. Panel (c) shows a failure case in which handwritten labels are incorrectly segmented as grooves. Additional failure modes include false positives near specular highlights and missed or fragmented masks for extremely small or weak-contrast grooves. These errors likely arise because high-contrast non-groove structures can produce intensity and gradient responses similar to groove boundaries, whereas very weak grooves provide insufficiently continuous evidence.

## 4. Conclusions

This study proposes PMW-YOLO, a lightweight instance segmentation network for automated detection of CED-ECM-processed shallow grooves. Through pseudo-multimodal channel fusion, the method constructs three complementary representations from a single grayscale image: original grayscale, CLAHE enhancement, and multiscale Sobel gradients. It also introduces DWTDown into the backbone to preserve boundary-related high-frequency components during early downsampling. Experiments show that pseudo-multimodal input provides more stable gains than the conventional RGB control on strict localization metrics and recall. DWTDown maintained baseline-level segmentation accuracy with 2.676 M parameters and 27.53 GFLOPs, indicating a modest complexity reduction that may be useful for resource-constrained inspection systems. Direct latency, memory, and edge-device tests are still needed before making a stronger deployment claim.

The repeated original-image-level holdout analysis shows that mAP gains from architectural or loss improvements such as EMA and boundary-aware loss are affected by small-sample variability. These gains are not yet sufficient to serve as stable sources of accuracy improvement, so this study reports them objectively as ablation results. Future work should expand the dataset and cover more machining parameters. It should also further address false positives from specular highlights and missed detections of extremely weak grooves, and evaluate online deployment capability by combining semi-supervised learning, test-time augmentation, and model quantization.

## Figures and Tables

**Table 1:** Ablation results for architecture and loss. Test set, 500 epochs, input size = 1024. mAP, P, and R are reported as fractions between 0 and 1.

Variant	Parameters	GFLOPs	Box/mAP50	Box/mAP50-95	Mask/mAP50	Mask/mAP50-95	P	R
baseline	2,689,079	28.46	0.9110	0.7191	0.8883	0.7357	0.912	0.855
+DWT	2,675,599	27.53	0.9058	0.7185	0.8976	0.7456	0.915	0.826
+EMA	2,702,743	28.95	0.9101	0.7035	0.9100	0.7281	0.940	0.862
+Boundary edge = 0.5	2,689,079	28.46	0.9045	0.7137	0.9145	0.7474	0.898	0.891
DWT + EMA + Bd	2,689,263	28.03	0.9138	0.7109	0.9118	0.7373	0.929	0.826
EMA + Bd edge = 0.1	2,702,743	28.95	0.9187	0.7159	0.9024	0.7359	0.937	0.865
EMA + Bd edge = 0.25	2,702,743	28.95	0.9113	0.7244	0.9087	0.7352	0.937	0.868

**Table 2:** Results from three repeated original-image-level holdout runs. Mask mAP50–95 and recall are reported as mean ± standard deviation (number of runs = 3). All metric values are fractions between 0 and 1.

Variant	Run-42	Run-1	Run-2	Mask mAP50-95/Mean ± Std	Recall/Mean ± Std
baseline	0.7357	0.7095	0.7330	0.7261 ± 0.0144	0.826 ± 0.033
+DWT	0.7456	0.6932	0.7397	0.7261 ± 0.0287	0.829 ± 0.005
+EMA	0.7281	0.7136	0.7327	0.7248 ± 0.0099	0.836 ± 0.026
+Boundary	0.7474	0.6917	0.7488	0.7293 ± 0.0326	0.864 ± 0.025
DWT + EMA + Bd	0.7373	0.6778	0.7369	0.7173 ± 0.0342	0.829 ± 0.012
EMA + Bd edge = 0.1	0.7359	0.7060	0.7129	0.7183 ± 0.0156	0.829 ± 0.032
EMA + Bd edge = 0.25	0.7352	0.6830	0.7308	0.7163 ± 0.0290	0.844 ± 0.025

**Table 3:** Control experiment for pseudo-multimodal fusion. Test set, 500 epochs; Δ = pseudo-multimodal—RGB. mAP, P, and R are reported as fractions between 0 and 1.

Model	Input	Box/mAP50	Box/mAP50-95	Mask/mAP50	Mask/mAP50-95	P	R
baseline	RGB control	0.9031	0.6932	0.9070	0.7216	0.916	0.873
baseline	Pseudo-multimodal	0.9110	0.7191	0.8883	0.7357	0.912	0.855
Δ (baseline)	-	+0.0079	+0.0259	−0.0187	+0.0141	−0.004	−0.018
bd_cp	RGB control	0.9142	0.7261	0.9126	0.7465	0.935	0.831
bd_cp	Pseudo-multimodal	0.9193	0.7305	0.9143	0.7578	0.934	0.899
Δ (bd_cp)	-	+0.0051	+0.0044	+0.0017	+0.0113	−0.001	+0.068

**Table 4:** Ablation of data-centric augmentation. The model is fixed as the boundary-loss variant, test set. mAP, P, and R are reported as fractions between 0 and 1.

Condition	Data	copy_paste	Box/mAP50	Mask/mAP50	Mask/mAP50-95	P/FP↓	R/FN↓
bd_base	Original	0.0	0.9045	0.9145	0.7474	0.898	0.891
bd_cp	Original	0.3	0.9193	0.9143	0.7578	0.934	0.899
bd_hn	Hard negatives	0.0	0.9146	0.9173	0.7452	0.904	0.870
bd_cphn	Hard negatives	0.3	0.9069	0.9071	0.7432	0.917	0.862

## Data Availability

The datasets generated and analyzed during the current study are available from the corresponding author upon reasonable request.
